# Adapting Pharmacoeconomics to Shape Efficient Health Systems en Route to UHC – Lessons from Two Continents

**DOI:** 10.3389/fphar.2017.00715

**Published:** 2017-10-10

**Authors:** Jacqui Miot, Michael Thiede

**Affiliations:** ^1^Department of Pharmacy and Pharmacology, Faculty of Health Sciences, University of the Witwatersrand, Johannesburg, South Africa; ^2^Scenarium Group GmbH, Berlin, Germany

**Keywords:** pharmacoeconomics, universal health coverage, efficiency, regulatory framework, Germany, South Africa

## Abstract

**Background:** Pharmacoeconomics is receiving increasing attention globally as a set of tools ensuring efficient use of resources in health systems, albeit with different applications depending on the contextual, cultural and development stages of each country. The factors guiding design, implementation and optimisation of pharmacoeconomics as a steering tool under the universal health coverage paradigm are explored using case studies of Germany and South Africa.

**Findings:** German social health insurance is subject to the efficiency precept. Pharmaco-regulatory tools reflect the respective framework conditions under which they developed at particular points in time. The institutionalization and integration of pharmacoeconomics into the remit of the Institute for Quality and Efficiency in Health Care occurred only rather recently. The road has not been smooth, requiring political discourse and complex processes of negotiation. Although enshrined in the National Drug Policy, South Africa has had a more fragmented approach to medicine selection and pricing with different policies in private and public sectors. The regulatory reform for use of pharmacoeconomic tools is ongoing and will be further shaped by the introduction of National Health Insurance.

**Conclusion:** A clear vision or framework is essential as the regulatory introduction of pharmacoeconomics is not a single event but rather a growing momentum. The path will always be subject to influences of politics, economics and market forces beyond the healthcare system so delays and modifications to pharmacoeconomic tools are to be expected. Health systems are dynamic and pharmacoeconomic reforms need to be sufficiently flexible to evolve alongside.

## Background

Every country is at some stage of using pharmacoeconomics to guide policy, financing, and other specific objectives in health system development. It is recognized that allocating resources across inputs into health systems, based on consideration of comparative efficiency, serves overall system efficiency. Pharmacoeconomics provides tools to pinpoint inefficiencies and to identify the most efficient therapy options. As a result, pharmacoeconomics has received increasing attention across health systems internationally.

Some countries have many years of experience in applying pharmacoeconomic tools and reasoning to determine pharmaceutical benefits, such as Australia, Canada, and the United Kingdom. Other countries have chosen a relatively narrow approach to applying pharmacoeconomic methods and use particular tools for decision-making in reimbursement and pricing of medicines in specific situations. This is the case in Germany and South Africa.

This paper explores factors that determine the introduction of pharmacoeconomics into health systems’ regulatory frameworks. Simultaneously, it seeks to provide guidance on processes for the design, implementation and optimisation of pharmacoeconomics as a steering tool within a health system under the universal health coverage (UHC) paradigm. The very distinct case studies of South Africa and Germany inform the identification of these determinants and guide the analysis of their influence on forming pharmacoeconomic policy and practice in different contexts. This unlikely selection of countries is based on the appreciation that their health systems have evolved along very different trajectories. The choice of pharmacoeconomic tools is determined by similar factors, however. The juxtaposition of the two scenarios therefore promises interesting insights with some generalisable lessons.

## Pharmacoeconomics in Germany; Principles and Evolution

Germany, a well-resourced, developed country runs a health system with a long history, shaped by the principle of self-governance, the realization of a “corporatist” approach that guides the entire German system of social protection. Social health insurance (SHI) is subject to the efficiency precept. This rule is set out in the German Social Code, stating that all services provided to SHI beneficiaries must be sufficient, appropriate, cost-effective and must not exceed the necessary. The Federal Joint Committee (*Gemeinsamer Bundesausschuss*, G-BA) is responsible for the maintenance of benefit packages according to the criteria of the efficiency precept. In the spirit of self-governance, it represents all relevant stakeholders, including medical and SHI physicians’ associations, hospital associations, associations of SHI funds as well as patients.

Under a broad definition of pharmacoeconomics, there are plenty of pharmaco-regulatory tools that constitute a challenging playing field for pharmacoeconomic research: At the macro level (with reference to the entire SHI pharmaceutical market) there are, *inter alia*, legislated mark-ups for pharmacies and wholesalers, and SHI discounts; at the meso level (with regard to groups of physicians and patients, specific therapeutic classes or Rx groups), group-specific individual prescription limits, regional target agreements, guidelines and discount contracts exist, as well as references prices; and at the micro level (for specific products, patients, physicians, or manufacturers) there are audits, prescription ceilings, second opinions and more. In order to understand the current German SHI regulatory framework and the (limited) role of the leading institute for the economic assessment of pharmaceuticals, some key health policy discussions need to be understood that have shaped the direction of pharmaceutical policy reforms.

The German reference pricing system has been in place since a major health reform changed the regulatory framework with effect from January 1989. The reference pricing mechanism in Germany works in several steps and respects the respective roles of the players within the system. Informed by technical experts, the G-BA is responsible for defining new reference price groups or changes in existing groups. Groups are formed within three distinct classes:

• Drugs with identical active ingredients (e.g., nifedipine);
• Drugs with pharmacologically and therapeutically comparable active ingredients (e.g., benzodiazepine-related products);
• Drugs with active ingredients that are therapeutically comparable (e.g., diuretics).

Within the classes, the grouping process takes into account different pharmaceutical formulations of products with specific active ingredients.

Reference pricing has been described as a “relatively blunt instrument for obtaining value for money from pharmaceuticals,” with health technology assessment (HTA) being a far superior strategy (Drummond et al., 2011). However, in Germany reference pricing has taken a core position within the set of pharmacoeconomic instruments in a broad definition of the term since 1989. Today, approximately 30,000 pharmaceutical products (counting different strengths and pack sizes) are subject to a maximum reimbursement price based on reference pricing. The National Association of Statutory Health Insurance Funds (*GKV-Spitzenverband*) estimates annual savings within the SHI system due to reference pricing at approximately 6.9 billion Euros^1^.

Among the various regulatory tools comprehensively discussed in the context of pharmaceutical regulation, the idea of introducing a positive list valid for the whole system ranked prominently at different points in time. The preparation on the basis of pharmacoeconomic considerations in the widest sense had already gone far, when the proposal failed spectacularly during a major health reform in 1992. Looking at other EU countries’ examples, the idea has been revived occasionally since but never again garnered much popularity in the relevant circles.

Since 2004, the Institute for Quality and Efficiency in Health Care (*Institut für Qualität und Wirtschaftlichkeit im Gesundheitswesen*, IQWiG) has taken on the role of a technically independent scientific institute with legal capacity. IQWiG’s responsibilities are

• to produce independent evidence-based reports on drugs, non-drug interventions, diagnostic tests and screening tests, clinical practice guidelines and disease management programs;
• to provide easily understandable health information for the general public;
• to conduct so-called early benefit assessments of new drugs; and
• to undertake health economic evaluations.

Usually, IQWiG’s projects are commissioned by the G-BA. Economic evaluation has been treated with utmost respect in the development of IQWiG’s methodological toolkit. The development involved a range of international experts who had a difficult time dealing with German stakeholders’ concerns around introducing more economics into the processes of defining and updating the benefit package. From inception, it took IQWiG 5 years to develop version 1.0 of its methods paper. IQWiG’s guidelines on economic evaluation sparked off more debate. The background section of the general methods stated intention “not... to develop a method enabling priority-setting within the health care system”; it also failed to properly address “value.” International health economists felt the need to point out the “sins of omission and obfuscation” in IQWiG’s guidelines on economic evaluation (Sculpher and Claxton, 2010). Particular attention was paid to the “efficiency frontier” approach, which limits evaluations to comparisons within a specific therapeutic area (Caro et al., 2010a,b; Dintsios and Gerber, 2010).

IQWiG’s methods have continuously been refined and improved over the years; version 5.0 was published in July 2017 after a period for comments on an earlier draft. The concept of the efficiency frontier is still being debated, refined and tested in policy practice and in health economic research (Mühlbacher and Sadler, 2017).

IQWiG has not developed a pharmacoeconomic routine as pronounced as that of NICE in the UK, for example. Yet with the Pharmaceutical Market Restructuring Act (*Arzneimittelneuordnungsgesetz*, AMNOG) in effect since January 2011, IQWiG has assumed a key role in the regulatory process of pricing pharmaceutical innovations. Here, IQWiG regularly conducts early benefit assessments on behalf of the G-BA based on dossiers submitted by manufacturers. The AMNOG process constitutes the first major effort to introduce price regulation for innovative non-reference priced pharmaceuticals upon market launch. Within this process, a pharmaceutical manufacturer must submit a value dossier to the G-BA upon market entry. Whilst the manufacturer may set the initial price freely, a price will be determined based on the additional benefit the product offers in comparison with an appropriate comparator. The so-called early benefit assessment will usually be commissioned to IQWiG. Based on the evidence and scope of an additional benefit, a discount on the originally set price will be negotiated between the National Association of Statutory Health Insurance Funds and the manufacturer, unless the product qualifies for a reference price group. The new maximum reimbursement price will come into effect 1 year after market entry. In its assessment of “value,” the AMNOG process ultimately considers a range of factors, including health gain, the share of patients benefitting, European price levels and the existence of generic comparators. Pharmacoeconomic research has not been able to establish the appropriateness of the resulting level of prices (Lauenroth and Stargardt, 2017).

Tying in the multiplicity of roles of players into the respective regulatory sets of tools, pharmaceutical regulation in Germany reflects the characteristics of the German politico-cultural context: firstly, the introduction of innovative instruments into the framework of pharmaceutical regulation happens slowly and requires political discourse and complex processes of negotiation; secondly, every instrument that gets introduced will be carefully grounded in the system’s history and its governance model. Overall, the approaches are results of negotiated processes following a coherent governance model.

## Pharmacoeconomics in South Africa; Evolution in Progress

At present, South Africa’s healthcare system is dichotomous with the public and private health sectors each providing services ranging from basic primary healthcare to highly specialized health services. However, there are considerable differences in that the majority of the population (84%) uses public healthcare which is largely funded through general tax revenue accounting for around 48% of total healthcare spend. A far smaller proportion (16%) access healthcare services through medical scheme insurance or out-of-pocket payments but this accounts for around 52% of healthcare spend in the country (Ataguba and McIntyre, 2012). The introduction of universal health coverage in the form of a National Health Insurance scheme for South Africa seeks to address these inequities.

The process of introducing UHC has its roots in the democratic reforms that occurred in the post-apartheid years from 1994. An initial version of National Health Insurance (NHI1), as described by van den Heever (2016) was envisaged in the period from 1994–2008 where pooling of resources within both the public (centrally pooled resource allocation) and private sector (risk equalization arrangement) was proposed. Subsequent to this, further reforms were proposed which replaced this with the concept of a single public entity (NHI2). At the heart of the proposal for NHI is the “principle of the Constitutional right of citizens to have access to quality healthcare services that are delivered equitably, affordably, efficiently, effectively and appropriately based on social solidarity, progressive universalism, equity and health as a public good and a social investment” (South African National Department of Health, 2017), Entrenched in this vision is the concept of introducing benefit design of packages of care using management and financing reforms such as rationing, strategic purchasing and contracting based on, amongst others, the principles of cost-effectiveness.

The development of a regulatory framework employing pharmacoeconomics was discussed for nearly a decade after the introduction of the concept in the National Drug Policy (NDP) of 1996 (South African National Department of Health, 1996). The Medicines and Related Substances Act 101 was amended in 2003 with the implementation of the Pricing Committee under Section 22G which would enable some of the reforms of the NDP (South African National Department of Health, 2003). The Regulations for a Transparent Pricing System, introduced in 2004, put into place the legal framework for pharmacoeconomic evaluation; however it was another 8 years before the first set of Pharmacoeconomic Guidelines was published in 2013 (South African National Department of Health, 2013; Gray and Suleman, 2015). The aim of these was to provide guidance on conducting and submitting a pharmacoeconomic analysis. The intention of the guidelines was to begin with voluntary submissions. Although the current regulations provide for a mandatory submission if so requested by the Director General of Health, this has to date, not been implemented. The pharmacoeconomic guidelines were not intended solely for the private sector and are applicable in the public sector setting as well.

Medicines selection processes in South Africa are probably the main area where pharmacoeconomics is currently being used in both sectors. In the public sector, the process for selection of medicines onto the Essential Medicines List (EML) allows for the use of pharmacoeconomics to assess the cost-effectiveness of proposed additions to the list. This has developed over time, however, with earlier editions of the EML only considering clinical evidence and some costing. Even in the current environment, full pharmacoeconomic analysis is lacking in areas (Perumal-Pillay and Suleman, 2017).

Pharmacoeconomic evaluation of medicines for selection to formularies or reimbursement policies is also carried out in the private sector. The regulations of the Medical Schemes Act 131, as amended, provide for a consideration of cost-effectiveness in the protocols and formularies developed for managed care. In its current Prescribed Minimum Benefit Review construct, the Council for Medical Schemes has included the requirement for cost-effectiveness assessment (Council for Medical Schemes, 2016). Some medical schemes and managed care organizations are already conducting pharmacoeconomic evaluations although the exact scope of this is unknown (Hofman et al., 2015). This too has evolved over time with earlier attempts using simple costing or cost-minimisation approaches (which are often still the mainstay of decision making) although there is increasing evidence of more established pharmacoeconomic analysis being carried out in this sector (Fraser et al., 2016).

While pricing regulations have enforced a limit on annual single exit price (SEP) increases for medicines in the private sector, the entry price to the market is determined almost solely by the manufacturer. The fair value price of these medicines at this entry point could be determined by pharmacoeconomics where there is recourse to enforce the provisions of the pricing regulations. However, once these medicines are on the market other factors come into play including extensions of patents and delays in the introduction of cheaper generics or biosimilars which influence pricing of medicines. The pricing regulations provide for a process of international benchmarking of medicine prices in the private sector against a basket of countries (Australia, Canada, New Zealand, Spain and within South Africa) and draft regulations were proposed in 2014 (Suleman and Gray, 2017). Multiple gazettes have been published and commented on since then and this will continue to be debated until finalized and implemented. The consideration of international prices is already taking place when a manufacturer applies for an SEP.

In the public sector, the process of competitive tendering for medicine contracts now also includes a review of international prices which has seen considerable success with the dramatic reduction seen in prices of medicines such as the anti-infectives and TB medicines (Pharasi and Miot, 2013) However, despite the opportunity to negotiate lower tender prices based on global best prices, affordability remains an issue particularly for medicines that do not have generics or where the only alternative may be a clone of the originator and still priced well above alternatives available in other countries.

Central to the use of pharmacoeconomics in the selection of medicines is the ongoing debate around lack of cost-effectiveness thresholds (CET) in South Africa. This discussion has included nuances such as whether there should be different CETs for different sectors or diseases. In addition, recent, methodologically determined papers have presented thresholds for countries such as South Africa (Woods et al., 2016) providing greater confidence in their validity than the oft misquoted WHO 1-3 x GDP threshold which has now been retracted (Bertram et al., 2016). However increasingly there is a view that CETs are not the final answer and healthcare decisions are multi-factorial which can be determined more systematically through multi-criteria decision analysis (Thokala et al., 2016).

It is exciting times for South Africa where considerable changes to the healthcare landscape are underway and it is unclear as to the home of pharmacoeconomics in the future of UHC. It was previously suggested that it would lie within the structures of a nationalized health technology assessment (HTA) institution (Hofman et al., 2015). The proposed NHI implementation structures of Ministerial Advisory Committees for Health Technology Assessment and Healthcare Benefits suggest that this will be the case, although these will continue to function alongside the Essential Medicine List Committees. The EML is likely to be an integral component to the introduction of benefit package design under NHI and so the pharmacoeconomic tool of evaluating one specific medicine compared to others should continue to develop and become more comprehensive (Suleman and Gray, 2017). Substantial legislative reforms will be required in order to implement NHI, particularly in the National Health Act and so the regulatory framework for pharmacoeconomics is expected to evolve alongside these reforms.

## Conclusion

The debate around the ideal application and institutionalization of pharmacoeconomics in a UHC environment will always be ongoing. Healthcare systems are dynamic and never complete. To this end, regulatory frameworks for pharmacoeconomics need to be sufficiently flexible in accommodating these living systems but also provide clarity and direction. The introduction of pharmacoeconomics into regulation does not come as a single event but rather a growing momentum. Experience from the case studies shows that successful implementation starts from a modest concept with the potential of refinement and (modular) amendment. A reference framework is essential, such as the efficiency precept and the principle of self-governance in Germany, or a defining strategy, such as the National Drug Policy in South Africa, to guide regulation.

Delays in implementation as well as design modifications are not unusual, as pharmacoeconomics is subject to highly political decision-making processes with interests beyond health policy. As is the case with other steering structures and mechanisms, the dynamics of evolving pharmacoeconomics within the health system depends on windows of opportunity. The space for reform is often created by dedicated leaders, as has been the case for health reform projects in both study countries. Yet both countries’ experiences also show other factors that drive pharmacoeconomic progress. Pharmacoeconomics comes hand in hand with general trends of the economisation of policy and the marketisation of the health system. As a data intensive practice, the technological and economic possibilities to establish required systems favor the swift implementation of pharmacoeconomics, which, by the way, does not necessarily imply a reduction of costs or an opportunity to save money.

There is great potential for countries not only to learn from each other but also to collaborate, e.g., by pooling data, by sharing tools and databases, and jointly refining the toolkit.

## Author Contributions

JM and MT conceived, drafted and critically revised the work. JM and MT hereby agree to be accountable for all aspects of the work and give final approval of the version to be published.

## Conflict of Interest Statement

The authors declare that the research was conducted in the absence of any commercial or financial relationships that could be construed as a potential conflict of interest.

## Footnotes

1 www.gkv-spitzenverband.de
